# Deep Sequencing-Based Transcriptional Analysis of Bovine Mammary Epithelial Cells Gene Expression in Response to *In Vitro* Infection with *Staphylococcus aureus* Stains

**DOI:** 10.1371/journal.pone.0082117

**Published:** 2013-12-16

**Authors:** Xiao Wang, Lei Xiu, Qingliang Hu, Xinjie Cui, Bingchun Liu, Lin Tao, Ting Wang, Jingging Wu, Yuan Chen, Yan Chen

**Affiliations:** College of Life Sciences, Inner Mongolia University, Hohhot, China; Queen’s University Belfast, United Kingdom

## Abstract

*Staphylococcus aureus* (*S. aureus*) is an important etiological organism in chronic and subclinical mastitis in lactating cows. Given the fundamental role the primary bovine mammary epithelial cells (pBMECs) play as a major first line of defense against invading pathogens, their interactions with *S. aureus* was hypothesized to be crucial to the establishment of the latter’s infection process. This hypothesis was tested by investigating the global transcriptional responses of pBMECs to three *S. aureus* strains (S56,S178 and S36) with different virulent factors, using a tag-based high-throughput transcriptome sequencing technique. Approximately 4.9 million total sequence tags were obtained from each of the three *S. aureus-*infected libraries and the control library. Referenced to the control, 1720, 219, and 427 differentially expressed unique genes were identified in the pBMECs infected with S56, S178 and S36 *S. aureus* strains respectively. Gene ontology (GO) and pathway analysis of the S56-infected pBMECs referenced to those of the control revealed that the differentially expressed genes in S56-infected pBMECs were significantly involved in inflammatory response, cell signalling pathways and apoptosis. In the same vein, the clustered GO terms of the differentially expressed genes of the S178-infected pBMECs were found to comprise immune responses, metabolism transformation, and apoptosis, while those of the S36-infected pBMECs were primarily involved in cell cycle progression and immune responses. Furthermore, fundamental differences were observed in the levels of expression of immune-related genes in response to treatments with the three *S. aureus* strains. These differences were especially noted for the expression of important pro-inflammatory molecules, including IL-1α, TNF, EFNB1, IL-8, and EGR1. The transcriptional changes associated with cellular signaling and the inflammatory response in this study may reflect different immunomodulatory mechanisms that underlie the interaction between pBMECs and *S. aureus* strains during infection by the latter.

## Introduction

Bovine mastitis–inflammation of the mammary gland–is the most significant disease in dairy cattle with regard to frequency of occurrence, animal welfare, and economic cost, which is estimated to approach $2 billion annually in the US [1]–[2]. Mastitis threatens the income of farmers and the image of the dairy sector regarding issues related to animal welfare, milk quality and public health, which is of particular concern as the inevitable indiscriminate use of antibiotics in tackling cattle mastitis would eventually result in the irrational exposure of humans to sub-lethal doses of these antibiotic residues through milk consumption, resulting in the worsening of antibiotic-resistance problems associated with antimicrobial chemotherapy in humans [3].

*S. aureus* is a gram-positive pathogenic bacterium, largely responsible for mastitis in humans and cattle [4]. Although *S. aureus* infection can result in obvious clinical mastitis, it often evades immune response mechanisms to institute life-long subclinical chronic infections. This contributes in no small way to the growing interest in the studies of the involvement of *S. aureus* in bovine mastitis. Classically, *S. aureus* is considered an extracellular pathogen [5]. Many studies have, however, confirmed its ability to invade and survive in diverse cell types, including mammary epithelial cells, neutrophils, and macrophages [6]–[8].

The virulence of *S. aureus* strains is a factor of type and level of expression of virulence factors, which modulate host cell signalings and elicit transcriptional responses in immunological cells which otherwise are silent in the presence of non-virulent strains. The understanding of this virulence factor–dependent host cell/microbe dynamics in the mammary gland is still rudimentary, thereby necessitating further studies.

Bovine mammary epithelial cells (BMECs) produce milk and contribute significantly to the immunity of the mammary gland [9]. BMECs express many inflammatory mediators, such as cytokines and chemokines capable mobilizing appropriate defense strategies against invading pathogens [10] in a way reminiscent of other epithelial tissues like the intestinal and respiratory epithelial tissues where inflammatory responses have been demonstrated to mobilize neutrophils against microbial invaders [11]–[16].

In many cases, however, the pathogen can evade the host immune response, resulting in its survival and propagation in infected BMECs. This persistence can lead to a lengthy non-shedding subclinical phase in which S. aureus proliferates in the gland, ultimately resulting in the development of immunopathology that enables the dissemination of infection to other tissues and shedding from the host. The survival of the pathogen in the host cells is believed to be achieved through a diverse range of mechanisms including the inhibition of phagosome maturation and the suppression of key immune-regulatory pathways that mediate the host immune response to infection. Therefore, analysis of the BMECs transcriptome in response to S. aureus infection may offer a deeper understanding of the cellular processes governing pathogen-epithelial cell interactions and how modulation of these cellular pathways can result in pathology. Furthermore, identification of transcriptional markers of infection may enable novel diagnostics for mastitis, providing new tools for disease management.

On-going developments in mammalian genome resources and high-throughput deep-sequencing technologies continually provide improved methodologies for analysis of the gene expression changes induced in mastitis caused by *S. aureus* in vivo. DGE tag profiling allows one to identify millions of short RNAs and differentially expressed genes in a sample without the need for prior annotations [17]–[19]. Sequencing-based methods measure absolute gene expression and avoid many inherent limitations of earlier microarray-based assays [20]–[23]. In the current study, to avoid the variation of gene expression at the bovine individual levels influenced by age, sex, and individual variability [24], we used the Illumina Genome Analyzer platform to investigate the pBMECs response in detail after infection with three distinct S. aureus strains (S56, S178 and S36) which differ in their expression of virulence factors in vitro. Many changes in gene expression were observed in the infected pBMECs. Moreover, differentially expressed pBMECs transcripts were identified 4 h after infection with the *S. aureus* strains, allowing us to evaluate the early host response to this bacterium. These data add a novel layer of information regarding the complex bovine molecular pathways elicited upon distinct *S. aureus* strains infection and the role these pathways play in establishing the host immune response to mastitis.

## Materials and Methods

### Ethics Statement

In this study, Milk samples were collected from cows by trained veterinarians following standard procedures and relevant national guidelines in the BAIMIIAO farm, huhhot, China. The samples were collected specifically for this study, with the full agreement of the farmers who owned the animals. The study was approved by the Animal Care and Use Committee of Inner Mongolia Autonomous Region, China. All animal procedures were performed according to the guidelines developed by the China Council on Animal Care and protocol and approved by the Animal Care and Use Committee of Inner Mongolia Autonomous Region, China.

### Primary Bovine Mammary Epithelial Cell Culture

Fresh milk was collected from eight healthy Holstein cows in mid lactation having no clinical signs of mastitis and excluded its bacterial infection by conducting bacterial test. pBMECs were isolated from the bacterial-free milk and cultured by following the method described in Danowski et al [25] and Buehring [26]. Briefly, Milk (1000 ml) was defatted by centrifugation at 1850 g at 20°C for 15 min in four centrifuge cups (250 ml each) and skim milk was removed. Remaining total cell pellets were re-suspended in 25 ml pre-warmed (37°C) phosphate buffered saline (PBS) and pooled in pairs. After a second centrifugation step (1850 g, 15 min at 20°C), the two total cell pellets were resuspended in 25 ml PBS solution and filtered (Falcon Cell Strainer 100 µm, BD Biosciences, Bedford, USA) into one falcon tube. After centrifugation at 500 g for 5 min, the pellets were re-suspended in warm growth medium.DMEM/F-12 (GIBCO, Invitrogen, Beijing) supplemented with FBS (10%), mycillin (100 U), hydrocortisone (1 ug/mL), progesterone (1 ug/mL), transferrin (5 ug/mL), insulin (5 ug/mL), L-Gln (200 mmol), and EGF (10 ng/ml) (Hyclone and Sigma, USA) and cultured in a 25 cm^2^-tissue culture flask at 37°C in 5% CO_2_. During the growth period, the medium was changed every three or four days. After approximate two weeks, when the cells reached confluence, we split the cells by removing the medium, washing with 2 ml PBS, and addition of 2 ml Trypsin/EDTA. 1 ml Trypsin was removed after 1 min and cells incubated for 15 min at 37°C and 5% CO_2_. Then 5 ml DMEM/F-12 medium containing 10% FBS were added for inhibiting the enzyme activity. The cells were centrifuged in 10 ml DMEM/F-12 medium for 5 min at 500 g, and seeded in 7 ml medium in new 25 cm^2^ tissue culture flasks. Before the third passage all cells were re-suspended in freezing medium consisting of 70% DMEM, 20% FBS and 10% DMSO, and stored in liquid nitrogen. All experiments were performed with cells in the fourth passage.

**Figure 1:**
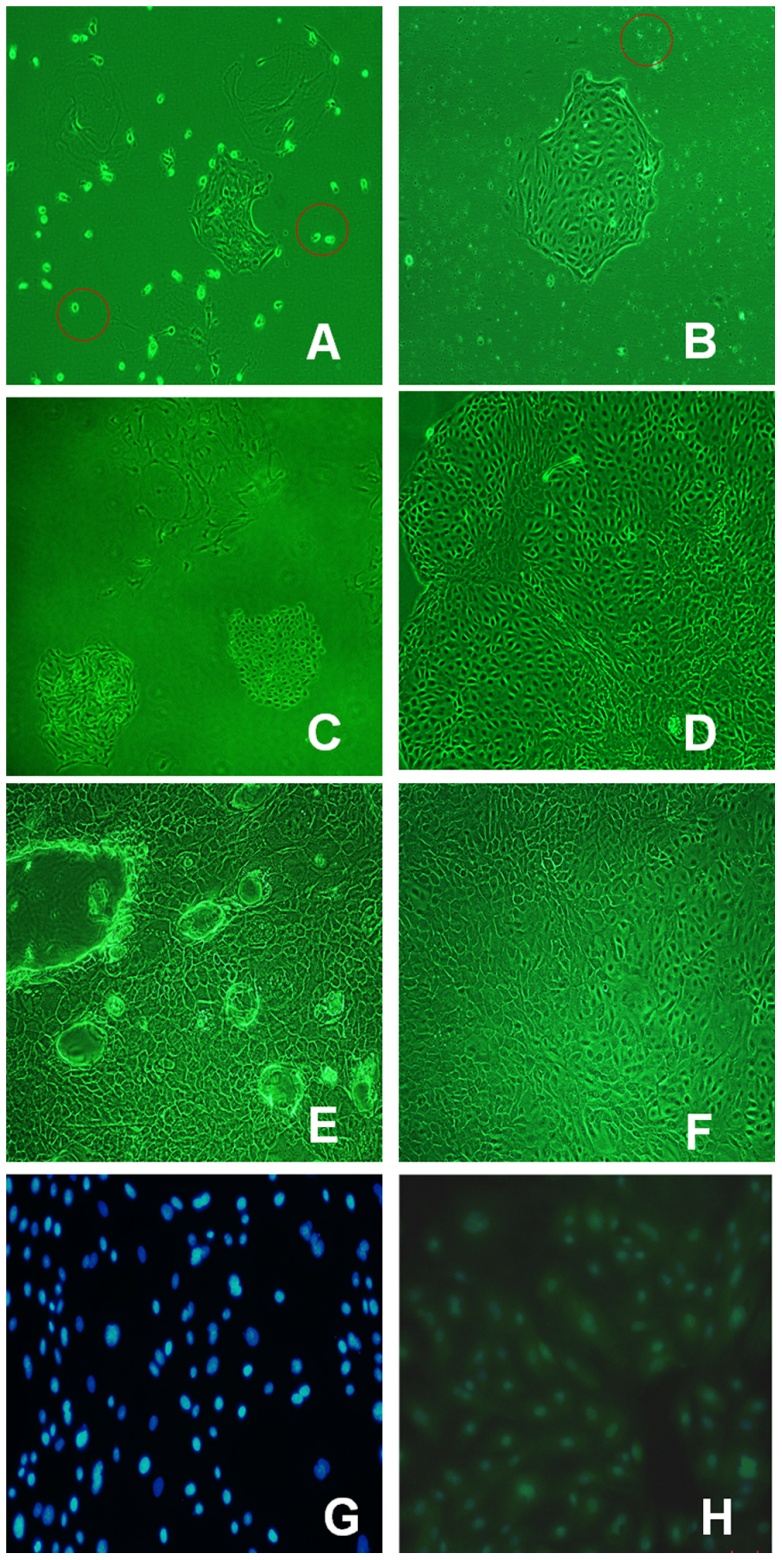
Primary bovine mammary epithelial Cell isolation and identification. Photomicrographs of the process obtained bovine epithelial cells and morphology of epithelial cells. (A–F). In panel A, single cells and small epithelioid islands of cells were apparent, spread upon the substrate after 24 h later, single cells with the granular appearance of macrophages were present. which noted by red color (100×); In panel B, a large epithelioid islands generated from a single cell after 3 days (100×). Occasional macrophages were present,which made up<5% of all attached cells. In panel C, many large epithelioid islands generated after 9 days, and phagocytes disappeared (100×); In panel D, areas of large cells arose within islands after two weeks in culture (100×); In panel E, the emulsion droplets secreted to the outside of the cells (100×); In panel F, the purified epithelial cells obtained over three passages (100×). Immunofluorescence analysis of bovine mammary cells (G-H). In panel G, first antibody (anti-cytokeratin 8) replaced by PBS as negative control, which pictured by fluorescence microscope. The cell nucleus were blue (DAPI) (100×).; In panel H, fluorescent image of cells incubated with anti-cytokeratin 8 monoclonal antibody, pictured by fluorescence (100×).

Kindly refer to figure 1, The epithelial origin and purity of pPBME were assessed by Immunofluorescence for keratin 8, a epithelial cell specific marker. To quantify the percentage of pBMECs, cells were counted under a fluorescence microscope, and five separate coverslips (three random fields per coverslip, 200 cells per field) were counted. The purity of all the cultures was assessed at >98%. In addition, to exclude leukocyte contamination, total RNA was extracted from the cells and transcripts of CD45, a leukocyte specific genetic marker, was not detected with the corresponding cDNA.

### Bacterial Strains and Growth Conditions

Three clinical isolates of *S. aureus* strains (S56, S36, and S178) were provided by Mr Yongqing Hao and Airong Zhang (college of veterinary medicine, Inner Mongolia Agricultural university, Hohhot). These clinical isolates were obtained from field-infected animals sourced from a herd with confirmed mastitis. All strains used were tested by RT-PCR for their expression of virulence factors. For this and for the cell culture experiments, bacteria were grown in brain heart infusion agar medium without shaking at 37°C for 12–14 h. They were subsequently inoculated into 5 ml of brain heart infusion fluid medium and incubated at 37°C with vigorous shaking for 4 h. The *staphylococcal* strains are listed in Table 1.

**Table 1:** *Staphylococcus aureus* Strains Used in the Present Study.

strain	number of invasiveness mean ± SEM	hemolysis	Properties	Reference or source
S56	34.93±6.34	+	Wild-type isolate from bovine mastitis MRSA	Blood culture isolate
S36	0.40±0.034	–	Wild-type isolate from bovine mastitis MRSA	Blood culture isolate
S178	10.00±3.46	+	Wild-type isolate from bovine mastitis MRSA	Blood culture isolate

**Note:** Different *S. aureus* strains were tested for invasiveness in pBMECs by a flow cytometry invasion assay. Results represent means ± standard errors of the mean (SEM) for 3 independent experiments performed in duplicate. After 4 h, hemolytic activity due to toxin expression was determined on columbia blood agar plates. Results are listed semiquantitatively in 3 categories: –, no hemolysis; +, effective hemolysis; ++, highly effective hemolysis. MRSA, methicillin-resistant *S. aureus.*

### Invasion Assay

PBMECs were washed three times with 1 ml/well DMEM. Thereafter, 1 ml of a suspension of *S. aureus* in DMEM was added per well. Cell monolayers infected with bacteria were incubated at 37°C in 5% CO_2_ for 4 h. After incubation, monolayers were washed three times with 1 ml/well of phosphate buffered saline (PBS, pH 7.4), supernatants were replaced with tissue-growth medium containing gentamicin (100 mg/ml) and incubated for 2 h at 37°C. After the incubation with cell-growth medium containing gentamicin, the supernatants were replaced 10 times in order to loosen any weakly adhering bacterial cells from the monolayer. The effectiveness of the antibiotic in killing extracellular bacteria cells was monitored by culturing supernatants from infected pBMECs treated with gentamicin. After cell-culture medium containing antibiotics was removed, pBMECs were washed three times with PBS, treated with 1 ml/well lysis buffer containing 0.25% trypsin and 0.25% Triton X-100 in PBS. Suspensions of bacteria for inoculation, cell-culture supernatants treated with antibiotics and PBMEC lysates were plated in duplicate on trypticase soy agar using serial 10-fold dilutions and incubated overnight at 37°C. The number of bacterial cells was determined by colony count.

### Transmission Electron Microscopy

PBMECs were grown in 6-well culture clusters and inoculated with strains of *S. aureus*. At 3 h after inoculation, monolayers were washed 3 times with PBS, and monolayers were fixed in 2.5% glutaraldehyde in 0.1 M phosphate buffer overnight at 4°C. Monolayers were washed 3 times with 0.1 M phosphate buffer (pH 7.2). Cells were scraped into 1.5 ml centrifuge tubes, centrifuged at 800 rpm for 10 min, and washed twice with PBS, and fixed in 3% glutaraldehyde, stained in 1% osmium tetroxide, and embedded in epoxy resin in the culture dish in situ. Electron micrographs were obtained using imagingplate technology (Ditabis, Pforzheim, Germany).

### *In vitro* Infection of pBMECs with Three Distinct *S. aureus* Strains and Total RNA Extraction

Monolayers of cells, prepared in 24-well tissue culture plates (Corning, Lowell, MA, USA), were washed twice with PBS, and 0.5 ml of DMEM was added. A total of 20 ul of bacterial suspension (1×10^8^ CFU/ml) was used for infection. The same volume of PBS was the control. Following infection, the cells were co-cultured with *S. aureus* strain at 37°C in 5% CO_2_ at a multiplicity of infection of 100 or left untreated as the negative control. At the end of the incubation, cells from the 3 independent tests were used for RNA extraction [27]. Total RNA was isolated using a kit per the manufacturer’s protocol. Total RNA was re-suspended in RNAse-free water and eluted from oligo magnetic beads in TE buffer (pH 8.0) at 95°C [28].

### DGE Tag Preparation

mRNA was enriched from the total RNA on oligo (dT) beads and reverse-transcribed. The cDNA was digested with NlaIII and ligated with Illumina adaptor 1. Mme I was used to digest 17 bp downstream of the CATG site, and the Illumina adaptor 2 was ligated at the 3′ end. After linear PCR amplification, the fragments were purified by gel electrophoresis followed by Illumina sequencing. Image analysis, base calling and quality calibration were performed using the Solexa Automated Pipeline, after which the raw data (tag sequences and counts) were deposited in the SRA (http://www.ncbi.nlm.nih.gov/sra/) database under submission number SRP029201.

The sequencing-obtained raw image data were transformed by base calling into sequence data, called raw data or raw reads. The raw sequences had 3′ adaptor fragments, low-quality sequences, and several types of impurities. Raw sequences were transformed into clean tags after various datprocessing steps.

### Gene Expression Annotation

To map the DGE tags of the total RNA, we created a virtual library that contained all possible CATG sites and 17-base reference gene sequences [29]. All clean tags were mapped to the reference sequences, and only a 1-bp mismatch was considered. Clean tags that mapped to reference sequences from multiple genes were filtered. The remaining clean tags were designated unambiguous clean tags. The number of unambiguous clean tags for each gene was calculated and normalized to the TPM (number of transcripts per million clean tags) [28], [30]–[31].

### Screening of Differentially Expressed Genes (DEGs)

To compare the differences in gene expression between samples (C/S56, C/S36, C/S178, S56/S36, S56/S178, S36/S178), the number of raw clean tags in each library was analyzed statistically as described [32]. With reference to Claverie et al. [32], we have developed a rigorous algorithm to identify differentially expressed genes between two samples. The p value corresponded to the differential gene expression test, and the false discovery rate (FDR) was used to determine the threshold of p values in multiple tests and analysis through manipulating the FDR value. A p-value <0.005, FDR ≤0.01, and estimated absolute log2-fold change >0.5 were set as the threshold of significant difference in gene expression [33].

### Cluster Analysis of DEGs Patterns

Genes with similar expression patterns usually correlate functionally. We perform a cluster analysis of gene expression patterns with the cluster [34] and Java Treeview [35] programs.

### Gene Ontology Functional Enrichment Analysis for DEGs

GO is an international standardized functional classification system that provides a dynamic, updated, controlled vocabulary and a strictly defined concept to describe the properties of genes and their products in any organism comprehensively. GO has 3 ontologies: molecular function, cellular component, and biological process.

In the gene expression profiling analysis, GO enrichment analysis of functional significance applied a hypergeometric test to map all differentially expressed genes to terms in the GO database, looking for significantly enriched GO terms in DEGs compared with the genomic background and calculated as follows:
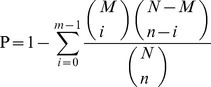
where N is the number of all genes with a GO annotation, n is the number of DEGs in N, M is the number of all genes that are annotated to certain GO terms, and m is the number of DEGs in M. During gene ontology analysis, the gene sets with P≤0.05 should be deemed significantly enriched.

### Pathway Enrichment Analysis of DEGs

Different genes usually cooperate with each other to exercise their biological functions. Pathway-based analysis helps to further understand genes biological functions. Kyoto Encyclopedia of Genes and Genomes (KEGG) is the major public pathway-related database. Pathway enrichment analysis identifies significantly enriched metabolic pathways and signal transduction pathways in DEGs compared with the entire genomic background. The calculating formula of pathway enrichment analysis is the same as that in the GO analysis. Here, N is the number of all genes with a KEGG annotation, n is the number of DEGs in N, M is the number of all genes that have been annotated to specific pathways, and m is number of DEGs in M. Pathways with Q ≤0.05 were considered significantly enriched.

### Quantitative Real-time PCR Analysis

The cDNA of pBMECs was reverse transcribed using the TRIzol reagent according to the manufacturer’s protocol (Tiangen, Dalian, China). Real-time RT-PCR reactions were performed using SYBR Premix Ex Taq (Takara, Dalian, China) on an ABI Prism 7500 Sequence Detection System (Applied Biosystems, Foster City, USA). Glyceraldehyde-3-phosphate dehydrogenase (GAPDH) was used for normalization [36]. Sense and antisense primers for the genes are shown in Table 2. Each reaction volume was 20 ul, and the reaction conditions were as follows: 95°C for 5 min, followed by 40 cycles at 94°C for 15 s, 56°C for 15 s, and 72°C for 30 s. Relative expression levels were normalized to glyceraldehyde-3-phosphate dehydrogenase (GAPDH).

**Table 2:** Sense and antisense primers for qPCR.

Gene bank ID	Forward primer	Reverse primer	Product size (bp)
BC103310	GAACTTCGATGCCAATGC	TCATGGATCTTGCTTCTCAG	216
U95811	AATGCTGCTCCTGCTCCT	CACTTCCTGACCAGTCTTGA	196
M37210	AAGGAGAATGTGGTGATGG	TGTAGTAGCCGTCAGGTAT	270
DT816782	GGAGACAGGTTGGAATGC	TTGTGAGGTGGCTGAGAG	285
BC105484	TGAAGGACGAGGAGTATGA	TTGTTCTGGAAGTTGAGGAA	217
DY070408	CACTCCAATCTTCTTGACTG	TCCTGCTCTTCACTGCTA	259
BC123885	GAAGATGAGCAGCAGGTT	CATATCAACAGGCGAGGAA	207
BC119867	GAATGACACCTTCCTGGAT	AGGCAACGAGGATAATGG	182
BC102901	AAGAGGAGAAGAGCGACTT	CTACTTGCGTGGCTGAAG	300
BC116043	CCATAGCCAAGGAACTGAT	CAACAACATCCACCACAAG	294
BC103310	GAACTTCGATGCCAATGC	TCATGGATCTTGCTTCTCAG	216
BC123488	CTGGACTCTAACAACAACAC	GACGAGGAGGAAGATGAAG	257
XM_871059	AGGAGGTCAACCAACACG	TTCTTAGTCGCTCCACCTC	213
XM_600955	GGATCAGCAAGGACAGATG	TCGCACAGTAGGTAGAGTT	153
U95811	AATGCTGCTCCTGCTCCT	CACTTCCTGACCAGTCTTGA	196
AB100737	AAGACGCTGTGAGTGCTA	GCTTCTGCTGTTGCCTAG	212
D82025	CGAGGAAGAATGGTGGTT	GGCTTGATTGTAGGAGACT	286
BC120105	AGTGAAGACGGCATCTAAG	CGCTCTCATCCTCCATATC	299
BC154386	TCAGAGGAGGAAGAAGAGG	ATCCGTCAGATTCACCATC	199
BC149028	AGTGTGAACGGATGGAAG	GGCTCAGGACTGTCATTC	238
BC103346	CCTGACCTACCACGACAT	CCATTCCAATGATGCTCTTC	236
BC123885	GAAGATGAGCAGCAGGTT	CATATCAACAGGCGAGGAA	207
DV781287	GCCTTCCTAACTCAGTGAA	TAGTGTCGGCATTCCTTC	237
AB100737	AAGACGCTGTGAGTGCTA	GCTTCTGCTGTTGCCTAG	212
BC114669	TAGCAGATGTGAACGAGTC	AACTTGAAGAGGCGGAAG	190
NM_001109980	CAGCGTCTTCTTCAAGGAA	GAAGTAGCGGTTGAGCAT	298
BC116132	ATACGCCAGCCTCTACTT	CCTTCAGCACACTCTTCTT	218
BC102105	AACGCAACAAGGAGAACC	CTGGAGTCGCTGAACATAG	161
BC149225	AGCAGCAAGAGACAGTAGA	CGTGTTGGACAGACAGATT	192
BC142224	GCCTCCAACCTGTATGAA	ATCCATCCAACAAGTCAGT	160

## Results

### Characterization of *staphylococcal* Strains

**Figure 2:**
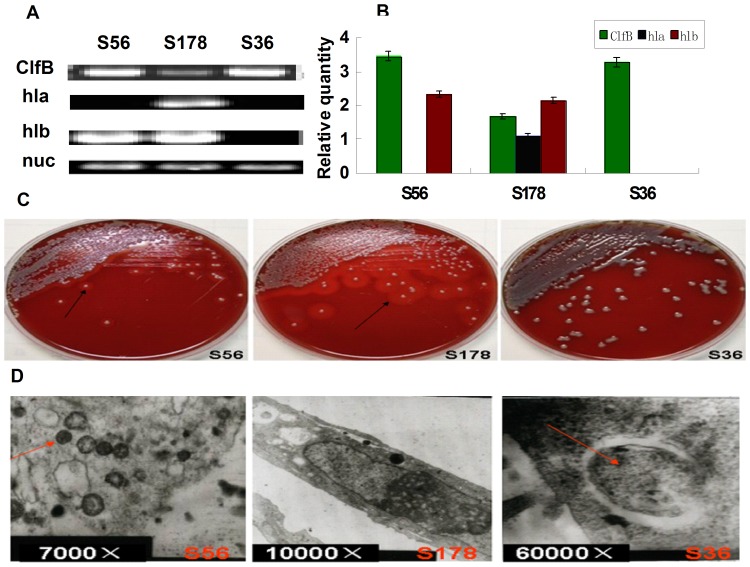
Characterization of 3 *staphylococcal* strains. S56: pPBME cells infected with S56 strain; S36: pPBME cells infected with S36 strain; S178: pBMECs infected with S178 strain. In panel A, *S. aureus* S56, S178 and S36 were grown overnight (as for the cell culture experiments), and bacterial RNA was extracted and analyzed by real-time polymerase chain reaction (PCR) for virulence factor expression (ClfB [Clumping factor B], hla [toxin gene], hlb [β-toxin] and nuc [*staphylococcal* nuclease gene]). The panel B showed the relative increase or decrease in gene expression. Data are means standard deviations for 3 independent experiments performed in duplicate. The panel C showed the hemolysin identification on columbia blood plate medium of *S. aureus*. S56 have a small hemolysis circle, S178 have the largest hemolysis circle and S36 have no hemolysis circle. The panel D is the electron micrographs of pBMECs infected with *Staphylococcus* aureus. pBMECs were infected with S56, S178 or S36 and incubated for 4 h. PBMECs were then fixed and processed for electron microscopy. For S56 and S36 strains tested, bacteria were internalized by pBMECs, while for S178 strain tested, bacteria were rejected by pBMECs.

Previous work has demonstrated that most clinical *S. aureus* isolates exhibit a strong invasive phenotype and that pro-inflammatory and cytotoxic effects in mammary gland cells are largely dependent on the invasive properties of the infective strains. Therefore, we selected 3 *staphylococcal* strains (S56, S178 and S36) for our study. Bacterial internalization was quantified using invasion assay and revealed that higher uptake of the S56 and S36 strains by pBMECs 4 h after infection, while no uptake of the S178 strains by pBMECs (Table 1). These results were confirmed by electron microscopy (Figure 2). For S56 and S36 strains, we detected intact staphylococci within the cells to further characterize *staphylococcal* strains, we performed reverse-transcription PCR to quantify the expression of important virulence factors, including adhesins and secreted toxins (Figure 2). The strain S56 efficiently expressed Clumping factor B and hemolysin (also confirmed by measurement of hemolytic identification on columbia blood plate medium) (Figure 2). In contrast, the wild-type isolates S36 efficiently expressed clumping factor B, but failed to express hemolysin. The wild-type isolates S178 strongly expressed hemolysin, but failed to express adhesins, accounting for the week invasive phenotypes.

### Analysis of DGE Libraries

To confirm the transcriptional changes in pBMECs, we analyzed the global changes in gene expression using the Solexa/Illumina DGE system, a tag-based transcriptome sequencing method. We generated 4 DGE libraries: 1 DGE library from the control group and 3 DGE libraries from every *S. aureus-*infected cells groups. The chief characteristics of these libraries are summarized in table 3. We obtained approximately 4.9 million total sequence tags per library and approximately 0.22 million distinct tag sequences. “Tags Containing N” means the number of tags that contain N of the total tags and distinct tags. The number of tags that contained N was approximately 0.015 million and 0.007 million. We filtered out the 3′ adaptor sequences, empty reads, low-quality tags, and tags with a copy number of 1, leaving approximately 4.8 million clean tags per library.

**Table 3:** Major characteristics of DGE libraries and tag mapping to the Unigenes transcript database.

	C		S36		S56		S178	
summary	Total	Distinct Tag	Total	Distinct Tag	Total	Distinct Tag	Total	Distinct Tag
Raw Data	4960570	229566	4855641	233775	4990174	280157	4728342	219562
Tags Containing N	14805	7473	12777	6869	13978	7736	14791	7429
Adaptors	304	255	284	245	347	281	321	269
Tag CopyNum <2	127384	127384	128780	128780	152860	152860	119205	119205
Clean Tag	4818077	94454	4713800	97881	4822989	119280	4594025	92659
CopyNum > = 2	4818077	94454	4713800	97881	4822989	119280	4594025	92659
CopyNum >5	4664652	40314	4555181	41934	4619996	45500	4443290	39521
CopyNum >10	4564770	27131	4450019	28069	4501432	29857	4345051	26589
CopyNum >20	4430881	17986	4310879	18577	4348611	19422	4215562	17719
CopyNum >50	4164877	9733	4037575	10113	4056738	10343	3953415	9545
CopyNum >100	3882107	5740	3736056	5869	3740853	5902	3668799	5549
Tag Mapping	4818077	94454	4713800	97881	4822989	119280	4594025	92659
All Tag Mapping to Sense Gene	3443100	39095	3338206	39135	3333491	42552	3317654	38284
Unambiguous Tag Mapping to Sense Gene	2567012	34311	2496348	34252	2530829	36521	2495296	33653
All Tag Mapping to Anti-Sense Gene	605435	21212	598121	22484	632425	23953	568962	11721
Unambiguous Tag Mapping to Anti-Sense Gene	522790	19385	503670	20612	525539	21849	486577	19355
All Tag Mapping to Gene	4048535	60307	3936327	61619	3965916	66505	3886616	59429
Unambiguous Tag Mapping to Gene	3089802	53696	3000018	54864	3056368	58370	2981873	53008
Unknown Tag	206532	8827	198828	9154	238700	15783	165857	8244

**Note:** All Mapping represents the number of all tags mapped to the Unigenes virtual tag database, Unambiguous Mapping represents the number of unambiguous tags mapped to the Unigenes virtual tag database, and Unambiguous Tags indicates tags matched only to 1 gene.

**Figure 3:**
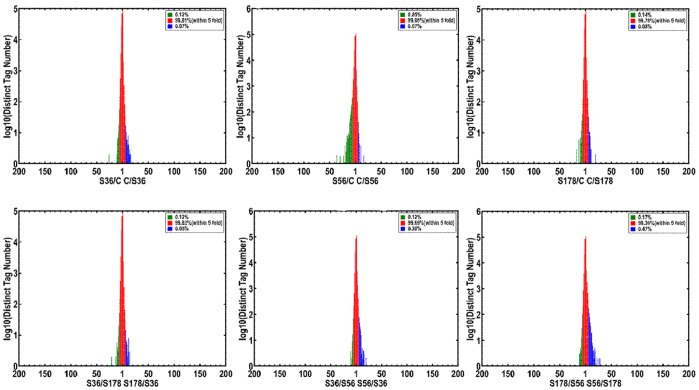
Distribution of the ratio of distinct tag copy numbers between the two libraries. S56: pBMECs infected with S56 strain; S36: pBMECs infected with S36 strain; S178: pBMECs infected with S178 strain; C: the non-infected pBMECs. Red means the distinct tags had ratios within 5-fold.

Heterogeneity and redundancy are 2 significant characteristics of mRNA expression. In analyzing the depth of the distribution of the ratio of distinct tag copy numbers between 2 libraries, we determined that the number of distinct tags within 5 was approximately 99.05% of the total distinct tags (Figure 3).

### Identification of DEGs Following Each *S. aureus* Strain Infection

**Figure 4:**
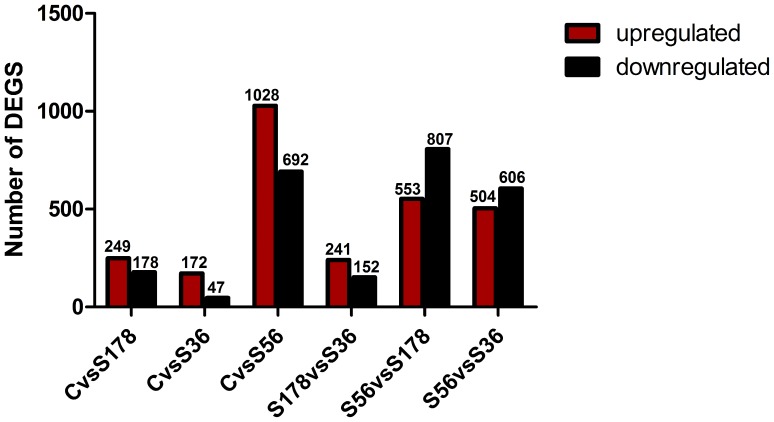
Differentially expressed genes between libraries. S56: pBMECs infected with S56 strain; S36: pBMECs infected with S36 strain; S178: pBMECs infected with S178 strain; C: the non-infected pBMECs. “FDR ≤0.001 and the absolute value of log2 Ratio ≥1” as the threshold to judge the significance of gene expression difference.

Initially, we compared gene expression profiles between C and S36 infection. Under the criteria of a P-value <0.005, FDR ≤0.001, and absolute log2 ratio >1 (Figure 4), the comparison of C vs S36 showed 15,901 transcripts, representing 219 unique genes were significantly differentially expressed. Of the 15,901 transcripts, 8963 (representing 172 unique genes) and 6938 (representing 47 unique genes) were upregulated and downregulated, respectively.

Likewise, the numbers of significant differentially expressed genes resulting from comparing C vs S56 and C vs S178 are summarized in Figure 4, which shows that S56-infected cells had the most differentially expressed genes and that S178 infected cells had the fewest.

### Identification of DEGs of pBMECs Infected with Different *S. aureus* Strains

Comparison of the gene expression profiles of S56 to S36 revealed 16,527 differentially expressed transcripts, comprising 1110 unique genes, with a P-value <0.005, FDR ≤0.001, and absolute log2 ratio >1 (Figure 4). Of the 16,527 transcripts, 8655 (representing 504 unique genes) and 7872 (representing 606 unique genes) were upregulated and downregulated respectively.

The results of the comparisons of S56 vs S178 and S36 vs S178 are also listed in Figure 4. For the differentially expressed genes between pBMECs infected with different *S. aureus* strains, S56 vs S178 had the most differentially expressed genes, while S178 vs S36 had the least.

**Figure 5:**
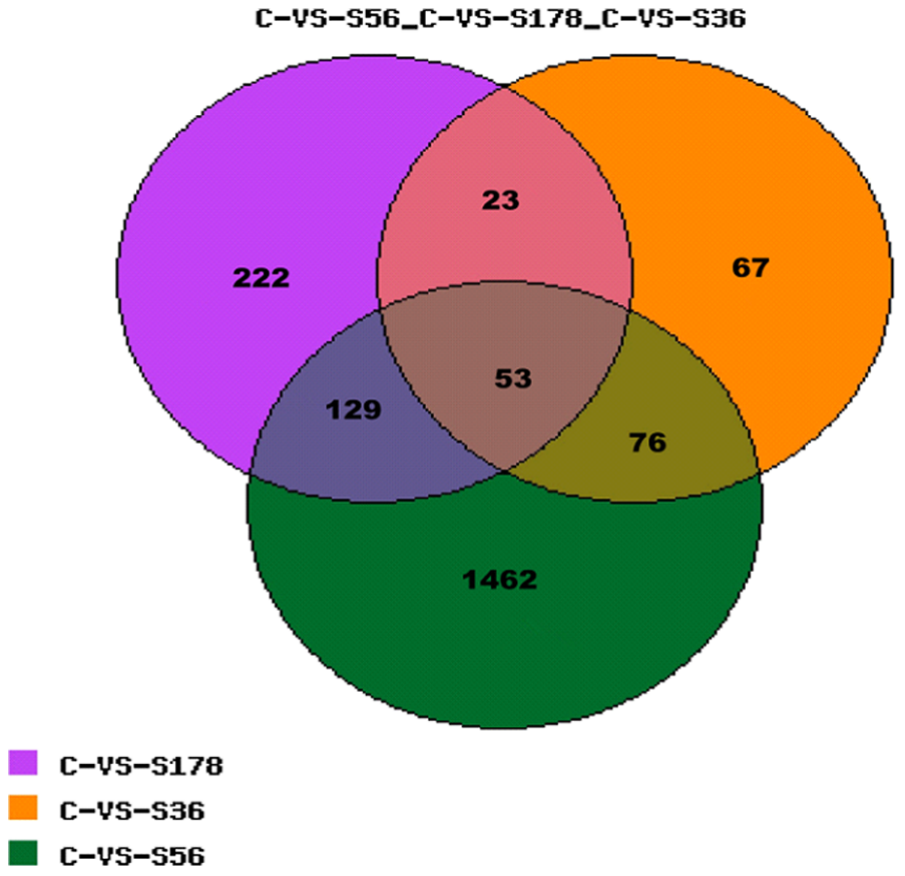
Venn diagram showing the number of differentially expressed genes overlapped in different comparisons. S56: pBMECs infected with S56 strain; S36: pBMECs infected with S36 strain; S178: pBMECs infected with S178 strain; C: the non-infected pBMECs. Number of genes overlapped among the three comparisons of C vs S56, C vs S178 and C vs S36.

**Figure 6:**
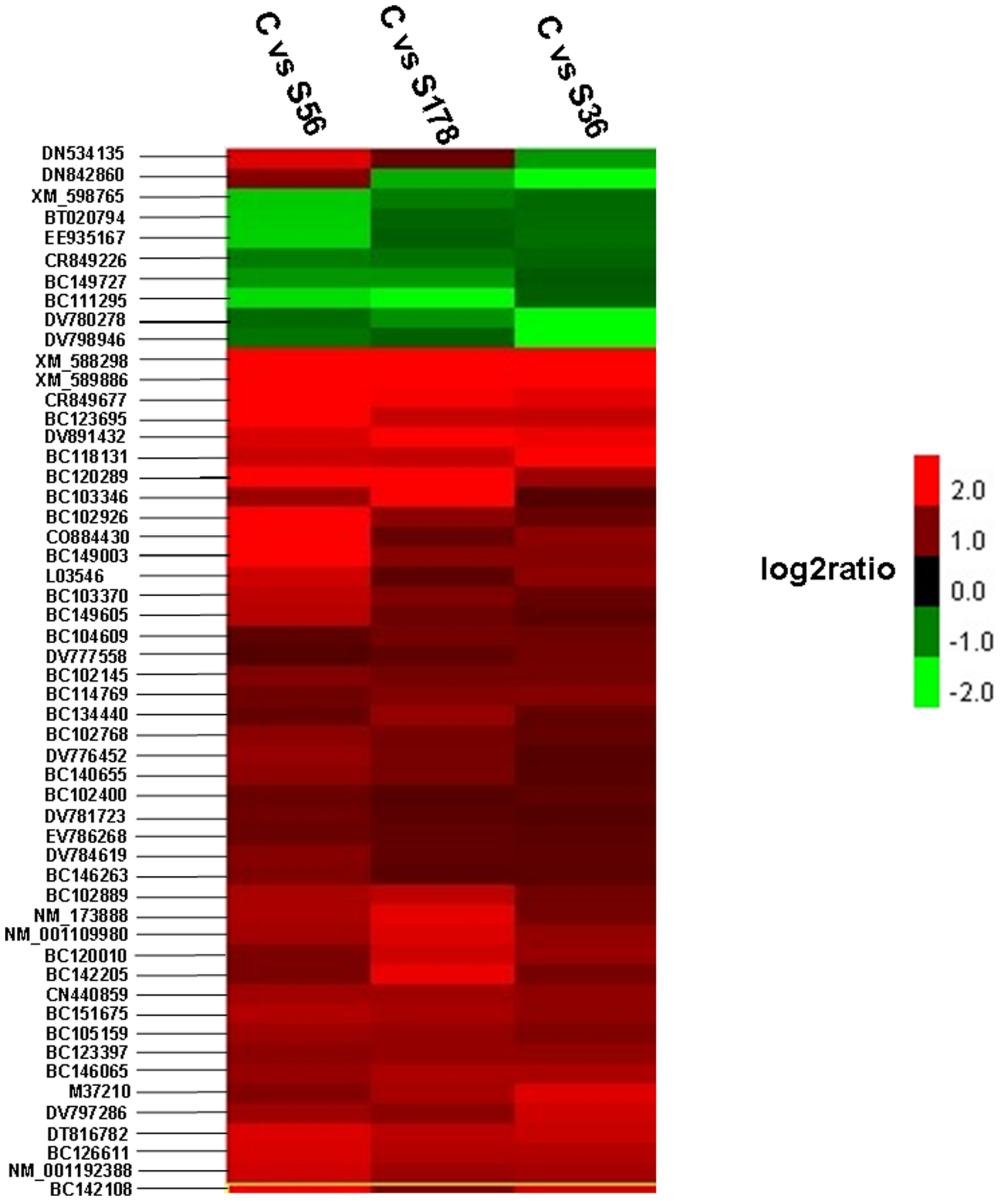
Clustering Analysis of Differential Gene Expression Pattern. S56: pBMECs infected with S56 strain; S36: pBMECs infected with S36 strain; S178: pBMECs infected with S178 strain; C: the non-infected pBMECs. Each column represents an experimental condition, each row represents a gene. Expression differences are shown in different colors. Red means upregulated and green means downregulated.

There were differentially expressed genes from the comparison of each infected pBMECs library with the control (i.e., C vs S36, C vs S56, and C vs S178), as well as from the comparison of pairs of infected libraries as shown in Figure 5. There were 53 differentially expressed genes from the three infected library-control pairs comparisons. Using the cluster [34] and Java Treeview [35] Programs 43 of the 53 DEGs were found to be up-regulated while 8 were found to be down-regulated. One gene was upregulated in the C vs S56 and C vs S178 comparisons but downregulated in the C vs S36 comparison. Conversely, one gene was upregulated in the C vs S56 comparison but downregulated in the C vs S178 and C vs S36 comparisons (Figure 6 and Table S1). The C vs S56 and C vs S178 comparisons generated the most differentially expressed genes, implying that infections with *S. aureus* S56 and S178 effect similar responses in pBMECs. The Genbank IDs of all genes are marked in Figure 6 and Table S1.

### GO Functional Enrichment Analysis of the DEGs between Infected and Non-infected Cells

We analyzed the functional effects of the gene expression changes between all pairs (C vs S56, C vs S178, C vs S36) by GO enrichment of DEGs, based on the GO database (Table S2). We focused on GO with a corrected-P value ≤0.05 (Table 4). Genes between C vs S56 and C vs S178 had similar biological processes with regard to metabolism, and C vs S36 have non common biological processes with C vs S56 and C vs S178. S56 and S178 modulated genes with similar molecular function and cellular components.

**Table 4:** GO Functional Enrichment Analysis of each *S.aureus* strain infection.

Terms for C vs S178_P			Terms for C vs S36_P			Terms for C vs S56_P		
GO term	Cf	P-value	GO term	Cf	P-value	GO term	Cf	P-value
response to abioticstimulus	25/273	1.00E-04	electron transportchain	10/130	7.68E-06	cellular metabolic process	549/1013	1.89E-05
de novo’ proteinfolding	8/273	1.11E-02	respiratory electrontransport chain	7/130	2.60E-04	metabolic process	636/1013	1.90E-04
cellular metabolicprocess	159/273	1.55E-02	cellular respiration	8/130	8.80E-04	primary metabolic process	539/1013	7.72E-03
metabolic process	182/273	4.25E-02	generation of precursormetabolites and energy	14/130	1.60E-03	ribonucleoprotein complexbiogenesis	35/1013	8.43E-03
response toradiation	14/273	4.80E-02	oxidation-reductionprocess	14/130	1.60E-03	cellular catabolic process	115/1013	8.97E-03
			response to acid	6/130	9.71E-03	cellular componentbiogenesis at cellular level	35/1013	1.89E-02
						cellular macromoleculemetabolic process	380/1013	2.00E-02

**Note:** The “Cf” means “Cluster frequency”, and in this column, the denominator represents the total number of genes and the numerator represents the number of each GO term genes.

### GO Functional Enrichment Analysis of the DEGs between Cells Infected with Different *S. aureus* Strains

We performed a GO functional enrichment analysis of S178 vs S36, S178 vs S56, and S56 vs S36 (Table S2); genes in S178 vs S36 and S56 vs S36 were enriched with regard to cellular respiration. In the cellular respiration and catabolism processes, S36 had the strongest influence on pBMECs. As shown in Table 5, genes from S178 vs S56 were not enriched in any GO function. No genes between S56 and S178 were enriched in terms of molecular function or cellular components.

**Table 5:** GO Functional Enrichment Analysis of different *S.aureus* strains.

Terms for S178vsS36_P			Terms for S178vsS56_P	Terms for S56vsS36_P		
GO term	Cf	P-value	GO term	GO term	Cf	P-value
cellular respiration	9/247	2.10E-02	none	cellular respiration	17/641	4.60E-04
regulation ofvasoconstriction	5/247	2.62E-02	none	respiratory electrontransport chain	11/641	1.97E-02
			none	regulation of cell cycle	40/641	2.86E-02
			none	cellular catabolic process	77/641	4.03E-02

**Note:** The “Cf” means “Cluster frequency”, and in this column, the denominator represents the total number of genes and the numerator represents the number of each GO term genes.

### Pathway Enrichment Analysis of the DEGs between Infected and Non-infected Cells

To examine the functional effects of gene expression changes that are associated with *S. aureus* infection, we performed a pathway analysis of DEGs, based on the KEGG database (Table S3).

Of all genes with a KEGG annotation, DEGs were identified in C vs S36. Ultimately, we assigned 136 DEGs to 120 KEGG pathways. We filtered those that were significantly enriched with a Q value ≤0.05. The enriched pathways included epithelial cell signaling in Helicobacter pylori infection, NOD-like receptor signaling, and chemokine signaling, which are linked to the immune response. Other pathways were oxidative phosphorylation, Parkinson disease, Alzheimer disease, Huntington disease, shigellosis, metabolism, osteoclast differentiation and Leishmaniasis, which are related to metabolism.

**Figure 7:**
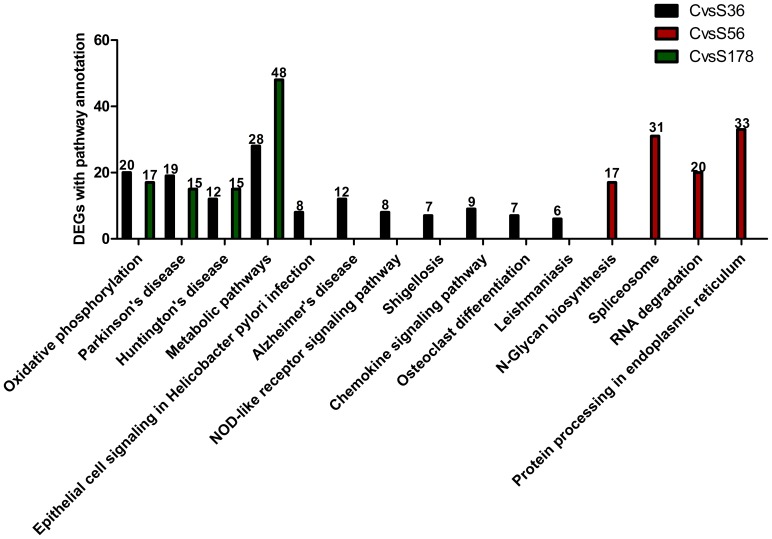
Pathway enrichment analysis of DEGs of each *S.aureus* strain infection. S56: pBMECs infected with S56 strain; S36: pBMECs infected with S36 strain; S178: pBMECs infected with S178 strain; C: the non-infected pBMECs. Pathways with Q value ≤0.05 are significantly enriched in DEGs. X-axis represents signaling pathways. Y-axis indicates the gene number.

The 1079 DEGs that were identified in C vs S56 were assigned to 214 KEGG pathways, 4 of which were significantly enriched. The 285 DEGs in C versus S178 were assigned to 170 KEGG pathways, 4 of which were significantly enriched (Figure 7).

### Pathway Enrichment Analysis of the DEGs between Cells Infected with Different *S. aureus* Strains

We analyzed enriched pathways in the DEGs between S56 vs S36, S178 vs S36, and S56 vs S178 (Table S3). The 721 DEGs in S56 vs S36 were assigned to 201 KEGG pathways, 4 of which were significantly enriched, including Parkinson disease, oxidative phosphorylation, N-glycan biosynthesis, protein export.

**Figure 8:**
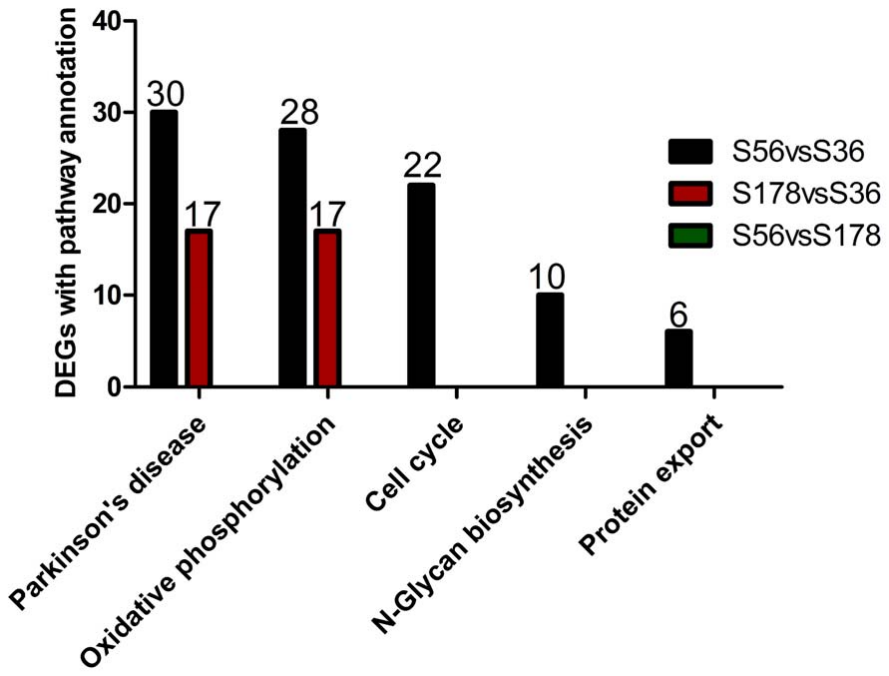
Pathway enrichment analysis of DEGs of different *S.aureus* strain. S56: pBMECs infected with S56 strain; S36: pBMECs infected with S36 strain; S178: pBMECs infected with S178 strain. Pathways with Q value ≤0.05 are significantly enriched in DEGs. X-axis represents signaling pathways. Y-axis indicates the gene number. There was no significant enrichment found by comparison of S56 vs S178, thus these comparison was not included in the figure.

The 249 DEGs in S178 vs S36 were assigned to 144 KEGG pathways, 2 of which were significantly enriched: oxidative phosphorylation and Parkinson disease. The 856 DEGs in S56 vs S178 were assigned to 211 KEGG pathways, but none was significantly enriched (Figure 8 and Table S3).

### Differential Expression of Immune-related Genes between *S. aureus*-infected and Uninfected Cells

**Figure 9:**
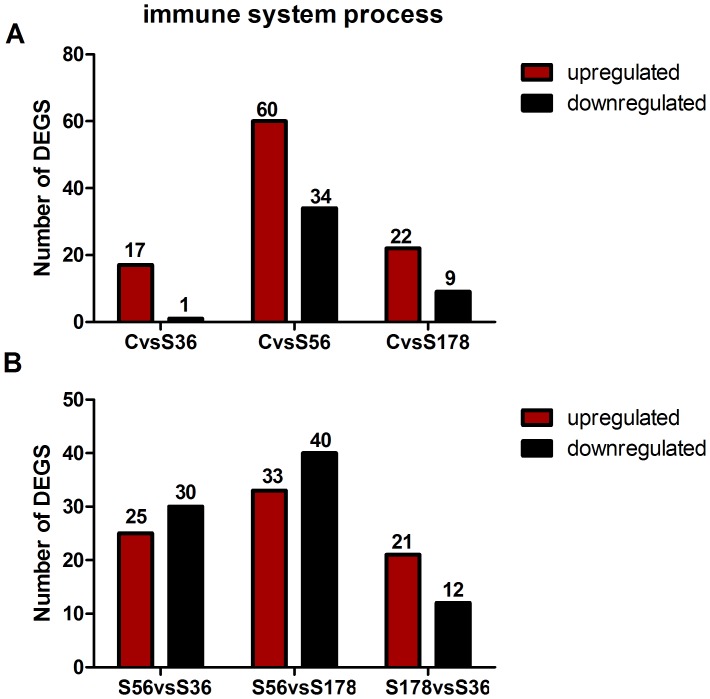
Differentially expressed of immune-related genes between the cells infected and non-infected with *S.aureus* strains. S56: pBMECs infected with S56 strain; S36: pBMECs infected with S36 strain; S178: pBMECs infected with S178 strain; C: the non-infected pBMECs. The panel A is the differentially expressed of immune-related genes between the cells infected and non-infected with *S.aureus* strains. The panel B is the differentially expressed of immune-related genes between the cells infected with different *S.aureus* strains. “FDR ≤0.001 and the absolute value of log2Ratio ≥1” as the threshold to judge the significance of gene expression difference. Red means upregulated and green means downregulated.

Due to the pathogenicity of *S. aureus* strains in pBMECs, immune-related genes are important for the host response to antigens [37]. The essential components of the innate immune response include proinflammatory cytokines, chemokines, and apoptosis-related genes [28], [38]. We analyzed the significantly differentially expressed immune- and disease-related genes between *S. aureus*-infected and uninfected cells (figure 9 and Table S3).

Of the 1720 unique differentially expressed genes between C and S56, 94 were immune-related, comprising 34 downregulated and 60 upregulated genes. The greatest upregulation was observed in Bos taurus mitogen-activated protein kinase kinase kinase 8, and Bos taurus ferritin heavy chain-like was the most downregulated.

Of the 427 unique differentially expressed genes between C and S178, 31 were immune-related genes–9 downregulated and 22 upregulated genes. The most upregulated gene was Bos taurus N-myc downstream regulated 1, and Bos taurus early growth response 1 was the most downregulated.

Of the 219 unique differentially expressed genes between C and S36, 18 (1 downregulated and 17 upregulated) were immune-related. The greatest upregulation was observed with IL-8, whereas Bos taurus early growth response 1 (EGR 1) was the most downregulated.

In C vs 56 and C vs S178, IL-8, Bos taurus chemokine (C-X-C motif) ligand 5, Bos taurus chemokine (C-X-C motif) receptor 4 (CXCR4) and bovine interleukin 1-alpha (IL-1-alpha) gene were up-regulated. Bos taurus insulin-like growth factor 1 receptor (IGF1R), Bos taurus virus-induced signaling adapter, and Bos taurus TNF receptor-associated factor 5-like were down-regulated between these pairs, but these genes are not found in S36 infected cells.

### Differentially Expressed Immune-related Genes between Infection with Various *S. aureus* Strains

Based on the differences in virulence of the *S. aureus* strains in pBMECs, we expected that certain immune-related genes would be differently expressed on infection between the strains (figure 9 and Table S3).

Of the 1110 unique differentially expressed genes between S56 and S36, 55 were immune-related; 30 were downregulated and 25 were upregulated genes. Bos taurus ephrin-B1 (EFNB1) was upregulated the most, and Bos taurus GATA-binding protein 3 was downregulated the most.

Of the 1360 unique differentially expressed genes between S56 and S178, 73 were immune-related; 40 were downregulated and 33 were upregulated genes. Bos taurus growth factor receptor-bound protein 7 was upregulated the most, and Bos taurus growth arrest and DNA-damage-inducible gene were the most downregulated genes.

Of the 393 unique differentially expressed genes between S36 and S178, 33 were immune-related; 12 were downregulated and 21 were upregulated genes. The greatest upregulation was observed with Bos taurus growth arrest and DNA damage-inducible factor; Bos taurus GATA-binding protein 3 was the most downregulated gene.

Bos taurus interleukin 8 (IL-8), Bos taurus ephrin-B1 (EFNB1), Bos taurus B-cell activating factor were upregulated in S178 vs S36 and downregulated in S56 vs S178 and S56 vs S36. Bos taurus cyclin-dependent kinase 6 was upregulated in S56 vs S178 and downregulated in S178 vs S36 and S56 vs S36.

### Validation of DGE Data by q PCR

**Figure 10:**
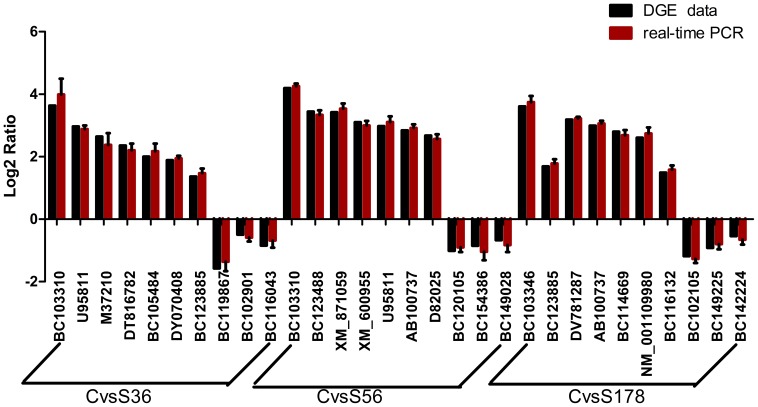
Expression pattern validations of selected genes by q PCR. Changes in transcript levels of 30 selected genes as detected by q PCR. The set included three downregulated genes and three upregulated genes. X-axis shows differentially expressed genes. Red bar indicates transcript abundance changes calculated by the DGEs method. Green bar with associated standard error bar represents relative expression level determined by q PCR.

To validate the DGE data that were identified by Solexa sequencing, we selected 30 genes for qPCR confirmation: 7 upregulated gene and 3 downregulated gene from the three comparisons of C vs S36, C vs S56 and C vs S178. As shown in figure 10, the genes that were differentially expressed in the DGE data were consistent with the real-time RT-PCR results, confirming the reliability of our DGE data.

## Discussion

In the present study, genome-wide expression profiles of three libraries of pBMECs, each infected with one of three *S. aureus* strains (S56, S178, and S36), were examined using the Solexa/Illumina digital gene expression system, a novel tag-based high-throughput transcriptome deep sequencing method. Differences in gene expression profiles between each library of infected cells and the non-infected control, as well as between the three infected libraries were measured. To the best of our knowledge, this is the first report of a remarkable strain-directed infection response of pBMECs to the afore-mentioned *S. aureus* strains.

**Figure 11:**
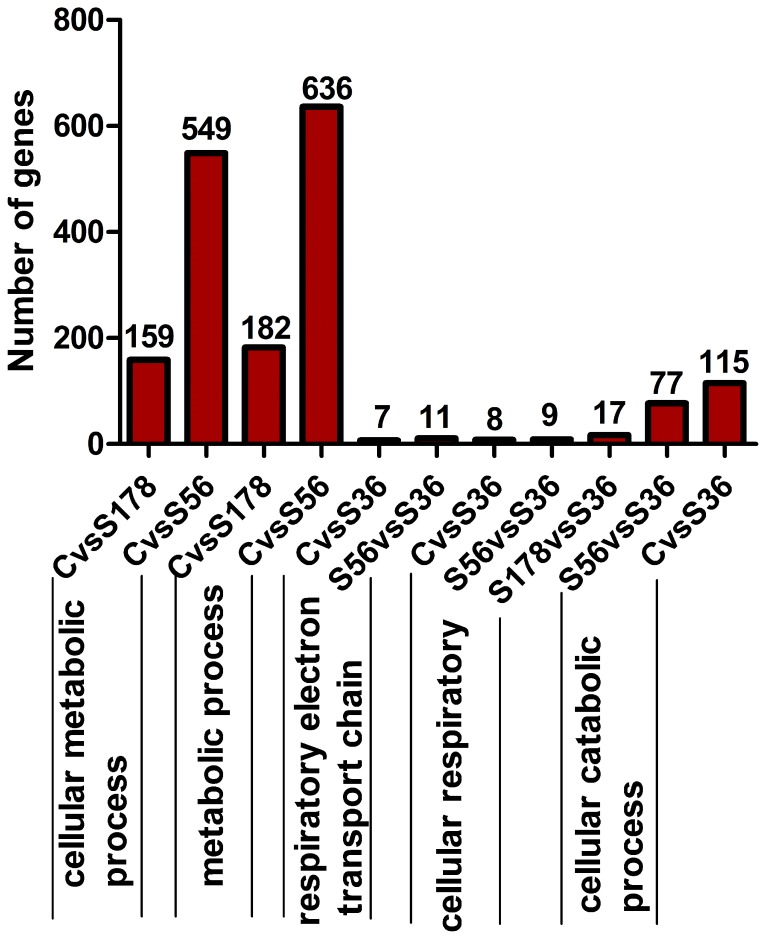
GO Functional Enrichment Analysis of different *S.aureus* strains. S56: pBMECs infected with S56 strain; S36: pBMECs infected with S36 strain; S178: pBMECs infected with S178 strain; C: the non-infected pBMECs. X-axis represents biological process. Y-axis indicates the gene number.

Infection with S56, S178, and S36 yielded 1720, 427, and 219 differentially expressed genes in pBMECs respectively (Figure 4). Though different in many respects, the DEGs yielded by the three test groups were shown by GO functional enrichment analysis of the changes in expression (Figure 11) to ultimately produce similar effects on cell cycle progression. This is an indication that infections by the three *S. aureus* strains affected cell cycle progression most probably by the expression of similar or somehow related toxins [39]–[40]. Notable in this regard is the similarity in the cell cycle progression influence of the DEGs induced by the strains, possibly because both S56 and S178 express hemolysin.

The differentially expressed genes induced by *S. aureus* strains were found to be associated with a number of biochemical pathways implicated in important metabolism, signal transduction and pathophysiology. For instance, the metabolic pathway clustered by the the S36 strain-induced DEGs (KEGG) (Figure 7) plays important roles in the metabolism of the amino acids glutamate and glutamine, which, like glucose, are important primary nutrients for cellular functioning [41]. In the same vein, the DEG-clustered NOD-like and chemokine receptors signallings are important innate immune and inflammatory signal transduction pathways: NOD-like receptors belong to specific families of pattern recognition receptors, and play a pivotal role in the recognition of the intracellular ligands, NOD1 and NOD2. NOD1 and NOD2 are two prototypic NRLs which sense cytosolic presence of bacterial peptidoglycan fragments that escape from endosomal compartments, initiating a cascade of events leading to the activation of NF-kB and MAPK, cytokine production and apoptosis [42]–[43]. The prominent enriched pathways in the S36- and S178-infected cells were cell cycle-influencing metabolic pathways (KEGG) [44]. Their other enriched pathways include the oxidative phosphorylation pathway and pathophysiological pathways like those involved in Huntington and Parkinson diseases which are in a way associated with cellular metabolism and apoptosis [45]. In contrast, the enriched pathways of the S56-induced cells were mainly endoplasmic reticulum-based protein processing (KEGG) (Figure 7) which are also ultimately apoptosis-linked [46]–[47]. BMECs are pivotal to the activation of innate immunity, forming an important line of defense against pathogenic microorganisms [9], [10]. We found that numerous important immune-related genes were differentially expressed upon separate infection with each of the three *S. aureus* strains (Table S4) which is in agreement with reports by earlier investigators [48]–[49]. Similarly, our results confirm earlier reports regarding the upregulation of cytokines, chemokines and other inflammation mediators genes in pBMECs during *S. aureus* infections. For example, the 4 h postinfection GO analysis of the three Control-infected library pairs showed highly significant upregulations in the nitric oxide synthase (NOS) genes of the S178-, S56- and S36- infected pBMECs libraries relative to the control, with log2 ratios of 2.57, 0.14 and 1.03 respectively: Nitric oxide (NO) is produced in cells in response to infections and plays a vital role in the early innate immune response that kills pathogens [50]. The same analysis showed a significant upregulation of prostaglandin-endoperoxide synthase 2 (Ptgs 2) gene in the S56-infected library relative to the control (log2 ratio 0.19), and significant upregulation (log2 ratio, 1.88) and downregulation (log2 ratio, −1.63) of chemokine (C-X-C motif) ligand 2 (Cxcl2) gene in the S36- and S178-infected libraries respectively relative to the control.

Mammalian toll-like receptors (TLRs) are members of the pattern recognition receptor (PRR) family that plays a central role in the initiation of innate cellular immune responses and the subsequent adaptive immune responses to microbial pathogens [44]. However, in the current study, there was no TLRs expressed differentially after *S. aureus* infection, and this might be a contributing factor to the molecular mechanisms underpinning the escape strategies of this pathogen from the mammary immune defence mechanisms to institute subclinical mastitis.

As noted earlier, more immune-related genes were altered by the infection of pBMECs with *S. aureus* S56 than with S36 and S178, for reasons not unconnected with virulence factors: Our results showed that the S56 strain is the most virulent of the three on the basis of its invasiveness and hemolysin expression (Figure 1). This trend is also seen to be consistent with the well established direct correlation between virulence gene expression and endothelial/epithelial cells attachment [51]–[54]. Summarily, these results portend that more genes alteration are required in the upregulation of immune, wound and inflammatory responses which characterize the more virulent S56 infection than in the cell cycle progression modulation that largely characterizes the less virulent S36 and S178 infections (Figure 11).

In conclusion, this report has described the evaluation of pBMECs trancriptome during infection with three *S. aureus* strains using a deep sequencing approach for DGE analysis, the outcome of which are divergent arrays of responses to the different strains, in relation to variations in their virulence factors. These findings are capable of producing better understanding of *staphylococcus* immunity and resistance, and could possibly lay a template for the discovery of more effective treatments for bovine mastitis caused by *S. aureus*.

## Supporting Information

Table S1 **The differentially expressed genes in all of the three comparison pairs.**
(XLS)

Table S2 **GO Functional Enrichment Analysis of the DEGs between cells infected with different** ***S. aureus*** **strains.** The significantly enriched pathways were marked with a red border.
(XLS)

Table S3 **Pathway enrichment analysis of the DEGs.** The significantly enriched pathways were marked with a red border.
(XLS)

Table S4 **The differentially expressed immune-related genes.** The red was upregulated the most, and the blue was downregulated the most.
(XLS)
